# Multi-Source Knowledge Reasoning for Data-Driven IoT Security

**DOI:** 10.3390/s21227579

**Published:** 2021-11-15

**Authors:** Shuqin Zhang, Guangyao Bai, Hong Li, Peipei Liu, Minzhi Zhang, Shujun Li

**Affiliations:** 1 School of Computer Science, Zhongyuan University of Technology, Zhengzhou 450007, China; 2019107118@zut.edu.cn (G.B.); 2019107117@zut.edu.cn (M.Z.); 2 Institute of Information Engineering, Chinese Academy of Sciences, Beijing 100093, China; lihong@iie.ac.cn (H.L.); liupeipei@iie.ac.cn (P.L.); 3 School of Information Science and Technology, Yancheng Teachers University, Yancheng 224002, China; lisj@yctu.edu.cn

**Keywords:** IoT security, threat analysis, ontology, knowledge reasoning, inference rules

## Abstract

Nowadays, there are different kinds of public knowledge bases for cyber security vulnerability and threat intelligence which can be used for IoT security threat analysis. However, the heterogeneity of these knowledge bases and the complexity of the IoT environments make network security situation awareness and threat assessment difficult. In this paper, we integrate vulnerabilities, weaknesses, affected platforms, tactics, attack techniques, and attack patterns into a coherent set of links. In addition, we propose an IoT security ontology model, namely, the IoT Security Threat Ontology (IoTSTO), to describe the elements of IoT security threats and design inference rules for threat analysis. This IoTSTO expands the current knowledge domain of cyber security ontology modeling. In the IoTSTO model, the proposed multi-source knowledge reasoning method can perform the following tasks: assess the threats of the IoT environment, automatically infer mitigations, and separate IoT nodes that are subject to specific threats. The method above provides support to security managers in their deployment of security solutions. This paper completes the association of current public knowledge bases for IoT security and solves the semantic heterogeneity of multi-source knowledge. In this paper, we reveal the scope of public knowledge bases and their interrelationships through the multi-source knowledge reasoning method for IoT security. In conclusion, the paper provides a unified, extensible, and reusable method for IoT security analysis and decision making.

## 1. Introduction

With the development of the Internet of Things (IoT), the massive deployment of IoT devices and the popularization of IoT technology have facilitated people’s lives [1]. As many organizations transform their devices to adopt IoT-connected technologies, it is estimated that there will be more than 40 billion IoT devices by 2027, with the potential value of the IoT going up to USD 11 trillion by 2025 [2]. However, the hidden security issues of the IoT are gradually emerging. Recent years have witnessed some of the largest, most sophisticated, and most severe cyber attacks, such as the SolarWinds attack [3] and the NetSarang malicious code attack [4], which affected millions of consumers and thousands of businesses. The in-depth integration of informatization and industrialization, such as the industrial control network of the mining, electric, and chemical industries, or the internal network of government, military, finance, etc., have all gradually developed from mutually independent and closed networks to interconnected and open ones [5]. The traditional boundary between the internal and the external networks is gradually being blurred, which consequently introduces ubiquitous network security risks. The integration of industrial control devices and network technology into the IoT environment eliminates the internal and external security boundaries of the industrial environment. Industrial control network devices do not only have security risks such as those affecting the security of software and hardware on the transmission link and the blurring of network protection boundaries, but they are also exposed to new threats such as a stepping attack, data sniffing, blockage, and eavesdropping within the IoT Cloud platform service. Hence, IoT security is challenging. Attacks on IoT devices are becoming more intelligent and diversified. Meanwhile, with massive numbers of IoT devices accessing the Internet, the interaction of heterogeneous information and rapid changes in the network structure have further expanded the scope of attacks and have led the IoT environment to continuously generate new weaknesses and threats [6]. When new threats are encountered, traditional security intrusion detection and response technologies cannot adapt to the attacks faced by IoT security. Security Information and Event Management (SIEM) and Security Operation Center (SOC) [7] also have limitations. Their mechanisms are often built on heuristic and static attack signatures and cannot detect new variants of attacks [8]. However, IoT systems are vulnerable to new families of attacks that could exploit the attack surfaces of devices and their network protocols. The SIEM system has a lack of security considerations for protecting their heterogeneous and complex devices and systems. IoT security requires an effective method to intelligently respond to security intrusions.

A variety of intelligent reasoning technologies can be used to recognize threats to the IoT. For example, the reasoning technology and semantic-web technology based on ontology [9], and the text mining and malicious code detection technology based on Natural Language Processing (NLP) [10,11]. However, due to the heterogeneity and complexity of the IoT, it is very difficult to create procedures for global security status detection and threat awareness in the IoT system. There are still challenges to security management and threat analysis within the IoT system.

This paper proposes a multi-source knowledge reasoning method for IoT security. We integrate knowledge to solve the semantic heterogeneity of multi-source knowledge by analyzing the characteristics of the security knowledge base for the IoT. In addition, we model an ontology to describe the elements of IoT security threats and design inference rules for threat analysis. This method can perceive the security status within the IoT environment and automatically infers mitigations to improve the threat response capability of the system.

Our contributions in this paper are as follows:

- We extracted the relationship between the entries of the IoT security public knowledge bases for knowledge integration, and the relationship mapping link graph model is constructed to provide support for the assessment of threat elements that affect IoT security;
- An IoT security threat ontology framework is proposed to describe the correlation of threat objects. The framework expands the current knowledge domain of network security ontology modeling and can provide a wider sense of security status;
- This paper proposes a reasoning method based on the multi-source knowledge of IoT security, which can perceive highly vulnerable platforms in the IoT environment and automatically respond to threats.

The remainder of the study is organized as follows: Section 2 discusses the literature review. Section 3 presents in-depth information on the IoT security multi-source knowledge base. Section 4 presents information on the methodology used in developing the proposed framework. After this, we formalize the classes of the proposed ontology model. Section 5 presents several scenarios to further illustrate the feasibility and effectiveness of the proposed model. Finally, we conclude the whole paper in Section 6.

## 2. Related Work

At present, there has been a lot of basic research around vulnerabilities and latent threats. Network security experts can formulate defense strategies, skills, and operations by using public structured description language and public cyber security knowledge bases. The literature analysis conducted suggests that there are several initiatives providing support for IoT threat analysis, predominantly through the use of threat modeling [12,13,14], knowledge graph [15], and graph theory [16] technologies. Syed et al. [12] integrated heterogeneous data, knowledge models, and common network security standards for information sharing and exchange from different network security systems. They built a unified cyber security model called Unified Cybersecurity Ontology (UCO), which supports information integration and network situation awareness. Abbas et al. [13] applied the STRIDE threat modeling method to the smart autonomous vehicular system and smart home, which identifies and mitigates threats that may lead to phishing attacks. Huang et al. [14] employed the anonymity of blockchain to protect identity information in threat intelligence. This work used encrypted threat intelligence to construct a complete attack chain and used a blockchain-based threat intelligence sharing framework for correlation analysis. Kiesling et al. [15] designed an evolving cyber security knowledge graph by integrating and linking critical information from multiple public knowledge bases such as vulnerabilities, weaknesses, and attack patterns; they also provided use cases for vulnerability assessment and sample queries for intrusion detection. Tian et al. [16] used the graph theory to model the network structure based on the complex characteristics of the network structure on the Internet. They also calculated the security situation of network nodes based on threat propagation, which can quantify the boundary link relationship of security protection.

However, the current research has not formally described the relationship between the core concepts of IoT security, which are unable to define inference rules because the research on threat analysis has not yet been improved to the semantic level. In this paper, we integrate the heterogeneous, multi-source knowledge bases for cyber security and uniformly represent the data in a graph; this graph will provide support for the next work on knowledge inference through the context of semantic information from different knowledge bases.

Ontology is used to describe information objects and support the sharing and reusing of domain knowledge. In Computer Science, an ontology is a formal description of concepts and relationships for an application domain of the real entity. A central aspect is the sharing of knowledge and information with the use of a common vocabulary, as supported by the Resource Description Framework (RDF). Ontology modeling is a means to formally model the structure of a system, which is useful for Cyber Threat Intelligence (CTI) analysis. At present, researchers have developed the open ontology for the security of IoT ecosystem elements [17,18,19,20], relating them with existing security concepts, primitives, weaknesses, vulnerabilities, and practices [21,22,23,24]. Researchers have conducted in-depth research on CTI analysis and ontology modeling in cyber security. Jia et al. [17] built a cyber security ontology based on assets, vulnerabilities, and attacks; they proposed a practical method for constructing a cyber security knowledge graph, and inferred new rules based on the five-tuple model of the cyber security knowledge base. Rastogi et al. [18] designed a malware ontology called MALOnt, which contained concepts such as malware characteristics, attack behavior, and detailed information about the attacker. It supported the collection of intelligence on malware threats from different online sources, and built a knowledge graph framework based on MALOnt. Mozzaquatro et al. [23] proposed an IoT network security framework for knowledge reasoning. It integrated knowledge on known network security issues (e.g., vulnerabilities and known threats) and the corresponding preventive measures into IoTSec [19] ontology. It enabled the security system to automatically detect threats to the IoT and to dynamically propose or implement the appropriate protection services. Choi et al. [20] proposed a security service framework that could be used in the power IoT-Cloud environment by analyzing the security vulnerabilities of the power system in that environment and by modeling the security context ontology. The framework used smart meters as an example to create an attack scenario in the power IoT, and realized a security mechanism that could operate effectively in this environment. Si et al. [21] proposed a knowledge base model of network security situation elements. The model was based on elements such as vulnerability attributes (e.g., severity of vulnerability, access request, result type, and distribution), vulnerability objects (e.g., configuration errors, protocol vulnerabilities), and constructed the ontology of the network security vulnerability. Li et al. [22] proposed a general network security parameter classification architecture, which extended the vulnerability ontology in the host domain based on the type of inheritance relationship.

Nevertheless, most of the current ontology modeling of cyber security are focused on vulnerabilities, weaknesses, and attack patterns. The research that combines the attacker’s Tactics, Techniques, and Procedures (TTP) with vulnerabilities and weaknesses is still in the exploratory stage. Therefore, it is difficult to perform multi-step attack prediction, threat analysis, and the follow-up work. The ontology model proposed in this paper expands the current knowledge domain of cyber security ontology modeling. This model provides a broader awareness of security status and improves threat response capabilities.

## 3. IoT Security Multi-Source Knowledge Base

Massive amounts of security information are fragmented and scattered on the Internet. The public information security knowledge bases maintained by the security organizations MITRE and NIST have gradually been developed into well-known public industry standards in cyber security. IoT security research results can be shown and shared through a set of structured information security description standards and specifications. We data mined a set of these information sources in order to expand upon their defensive utility in threat analysis. Well-known public knowledge bases in cyber security that we used in our work include:

- Common Vulnerabilities and Exposures (CVE) [25];
- National Vulnerability Database (NVD) [26];
- Common Weakness Enumeration (CWE) [27];
- Common Attack Pattern Enumeration and Classification (CAPEC) [28];
- Common Platform Enumeration (CPE) [29];
- Adversarial Tactics, Techniques, and Common Knowledge (ATT&CK) matrix [30].

### 3.1. Data Sources

CVE discloses the exposed vulnerabilities. A vulnerability is defined as a “weakness in the computational logic (e.g., code) found in software and hardware components that, when exploited, results in a negative impact on confidentiality, integrity, or availability” [26]. Each vulnerability in the database has an identification number and a related description defined by MITRE. NVD provides information about security-related software vulnerabilities, product configurations, and impact indicators. NVD is built on the CVE list and is fully synchronized with it as well. NVD provides enhanced information for entries in the CVE list, such as structured information, severity score, and impact level given by the Common Vulnerability Scoring System (CVSS) [31]. CVSS is an industry public standard for evaluating the severity of vulnerabilities. Most vulnerability severity research and commercial vulnerability management platforms are evaluated based on CVSS. CAPEC provides a summary of the attack pattern classification and focuses on the attacker’s use of cyberspace vulnerabilities. Security managers who understand attack patterns are essential to threat analysis and defense. CPE entries are specifically of interest because cyber security managers can scan them in order to be alerted to specific targets in their IoT systems. “Weaknesses are flaws, faults, bugs, and other errors in software and hardware design, architecture, code, or implementation that, if left unaddressed, could result in systems, networks, and hardware being vulnerable to attack” [32]. Information on the weaknesses is summarized by the CWE.

### 3.2. ATT&CK Matrix

The ATT&CK matrix was first proposed by MITRE in 2013. Through the summarization and analysis of real observational data and Advanced Persistent Threats (APT) [33], ATT&CK has gradually developed into a general language for attackers’ behavior description and a behavior analysis model for the entire life cycle of the attack chain. ATT&CK abstractly describes a framework composed of sequential network attack tactics, each of which covers abundant attack techniques. From the perspective of attack detection and threat analysis, the context information associated with the attack can be further speculated only when the attack tactics and techniques are clearly defined. ATT&CK persistently constructs and enriches the attackers’ tactics and techniques in order to help researchers to master the global attack technique needed to support the assessment and automatically respond to security intrusions.

Researchers are currently focusing on the feasibility of applying the ATT&CK matrix to threat analysis. In security intelligence research, vulnerability intelligence, which is mostly from the perspectives of software, hardware, operating system, and protocol weaknesses, developed earlier and is more mature [34]. However, threat intelligence mainly collects external factors related to attackers or attack behaviors [35]. Security managers achieve timely management and control of threats by integrating threat information and facilitating the sharing threat information [36]. Apart from the inherent complexity of the IoT, the heterogeneous information exchange between IoT devices and systems further aggravates its structural complexity [37]. Researching on vulnerability intelligence has great limitations in the complex environment of the IoT. Therefore, researchers hope to conduct analyses of threats in the IoT environment by analyzing and understanding the attackers’ targets and systemic risks. The ATT&CK matrix can connect threat events and observation data, and can further open up the link to promote an understanding of the threats [38].

### 3.3. Knowledge Integration and Relationship Mapping

**Figure 1:**
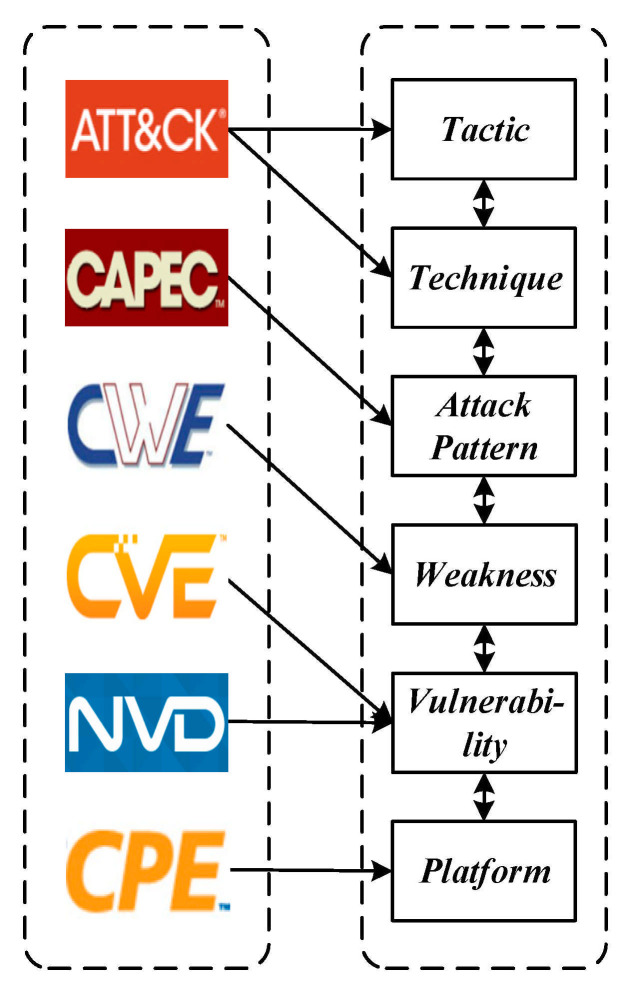
Schematic of relational mapping linkages.

It is worth noting that Attack Patterns in CAPEC connect the ATT&CK matrix to the CWE source, functioning as bridges that link a Technique within a Tactic to a CWE entry. Meanwhile, a CWE entry has a relational link to a CVE entry. The relationship implies that the Vulnerability is an example of the Weakness. In this paper, we use the Attack Patterns as bridges that relate a means of attack, i.e., Tactic and Technique, to its targeted Weakness. A Weakness in CWE can be linked to a Vulnerability in CVE, and it can be linked to a known affected Platform in CPE. Security researchers can select a particular application, hardware, or operating system in cyberspace to see which Tactics or Techniques will be affected by this end-to-end linkage method. Figure 1 illustrates the relational linkage mapping based on the selected knowledge bases.

Based on the mapped relationship links, this paper combines the source data and structural characteristics of each knowledge base, and uses a graph structure to uniformly represent the data. Each layer represents a different source. The nodes of the graph represent the entries of the knowledge bases. The internal and external links between the knowledge bases are retained and represented by the edges of the graph. These edges are not bi-directional in the source knowledge bases. However, when integrated into the unified graph structure data, it can be traversed bi-directionally and it is easy to trace the relationship between different knowledge sources from any node. In a nutshell, ATT&CK provides the Tactics and Techniques used by attackers on vulnerable systems. CWE, CVE, and CPE reveal the positions of Weaknesses and Vulnerabilities exploited by attackers in the IoT. Moreover, CAPEC associates potential attacks with Weaknesses that may become targets.

## 4. Ontology-Based Multi-Source Knowledge Reasoning Framework for IoT Security

Attacks on heterogeneous networks are the most prominent in the IoT environment [39]. The proposed method in the aforementioned section provides links to the understanding of threats and an overall view of the IoT security status. However, awareness of the network situation in multi-source heterogeneous IoT environments is a challenge. Ontology plays an important role in solving the semantic heterogeneity of CTI through the formal description of specific domain knowledge [40,41]. We use the Web Ontology Language (OWL) [42] to build a unified formal description. Concepts are implemented as classes, and relationships are implemented as properties. The expressive ability of OWL is limited to description logic and cannot express uncertain knowledge such as the changes of events in spatio-temporal and semantic relations. In order to enhance the reasoning ability of this model, the second half of this paper uses the semantic web rule language to design inference rules that complement the description ability of ontology. This paper proposes an IoT security knowledge reasoning model based on semantic ontology and rule logic. Our vision is to present a novel approach that improves IoT cybersecurity awareness of situation and focuses on the fusion of multi-source heterogeneous knowledge and the analysis of vulnerabilities, weaknesses, attack patterns, techniques, and tactics in a unified knowledge base. This approach will also enable the subsequent security service provisioning adjusted to the dynamic threat intelligence analysis, hence improving the security response mechanisms around threat intrusion and IoT assets.

**Figure 2:**
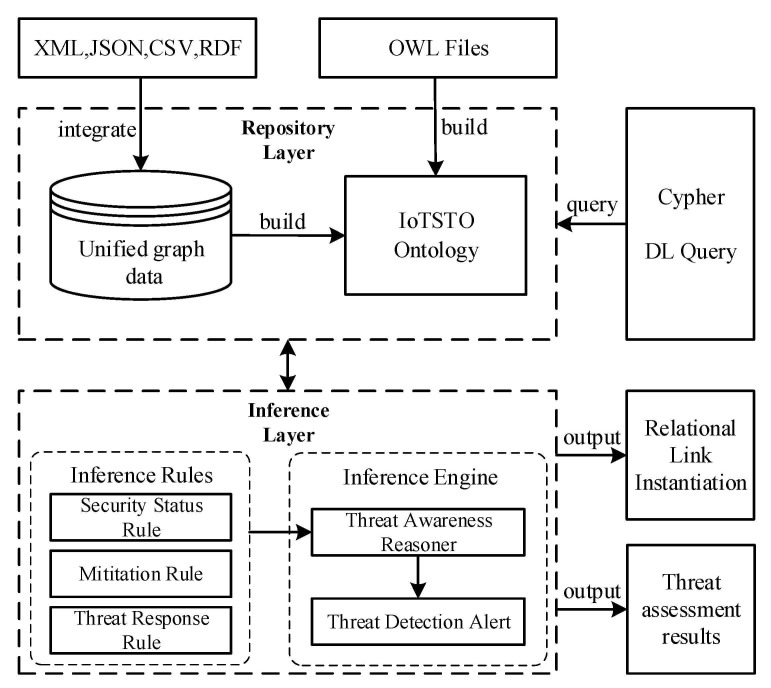
Multi-source knowledge reasoning framework for IoT security.

Figure 2 shows the multi-source knowledge reasoning framework for IoT security. The framework consists of a data and ontology repository and a reasoning engine. The heterogeneous data of multiple knowledge bases are preprocessed into unified graph data. The ontology model is constructed based on the integrated knowledge base characteristics and the external ontology model. In the inference layer, there are inference rules designed according to a specific IoT environment and applied to threat response modules. The workflow of the proposed framework is as follows:

1. The multi-source heterogeneous IoT security knowledge is obtained from crawlers embedded in several knowledge sources. The amount of knowledge is huge and the structure of the knowledge is different;
2. The crawled multi-source heterogeneous knowledge is integrated into a unified graph database;
3. The integrated data are mapped into the proposed ontology model through instance mapping, and the generated instances are integrated into the ontology repository;
4. The inference engine perceives and separates the abnormalities based on the instances repository and the user-defined inference rules to achieve the goal of automatically responding to threats.

We analyzed the characteristics of multiple knowledge bases of IoT security, and we proposed an IoT Security Threat Ontology (IoTSTO), which was inspired by UCO [12], IoTSec [19], and VulKG [43]. Furthermore, some concepts were extracted from these works, but with many details adjusted to make the ontology more suitable for knowledge bases. For example, we designed classes of tactics and techniques based on the ATT&CK matrix. The knowledge bases associated with these classes were not involved in the referenced ontology.

### 4.1. Classes and Attributes Analysis of IoTSTO

**Figure 3:**
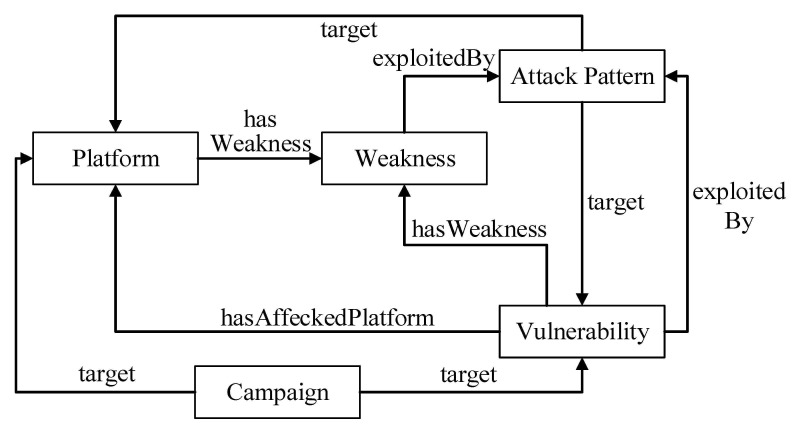
Top classes of IoTSTO.

An ontology is a major component of semantic technology used in the modeling of data. In this paper, the ontology model is used as a bridge that generates services by conducting the knowledge reasoning for multi-source heterogeneous IoT security data. We apply an ontology-based reasoning, which is required for clear decision-making and a quick response to threats occurring continuously in an IoT environment. As shown in Figure 3, IoTSTO includes five top-level classes: *Platform*, *Vulnerability*, *Weakness*, *Attack Pattern,* and *Campaign*. The classification of classes is based on the previously mentioned knowledge base hierarchy, but it is different. *Platform* describes the scene information of the IoT security event, including the software, hardware, and operating system that may be affected by the vulnerability. Meanwhile, *Platform* also contains the product vendor and version information. *Status* is a subclass of the *Platform*, which describes the vulnerability of the affected platform. *Campaign* describes a set of malicious activities or attacks that occur against a set of specific targets over a period of time. *Campaign* can be expressed by the tactics, techniques, resources (tools, malware), groups that issued the malicious activities, and the mitigations that defend the system against this campaign.

Table 1 shows the level 2 and level 3 class definitions in the IoTSTO. The following paragraphs are based on the top-level classes of the IoTSTO and introduce various ontology descriptions of the sub-domains.

#### 4.1.1. Ontology Description of the Platform

The *Platform* class includes the software, hardware, and operating systems affected by threats in the IoT environment, and there are corresponding sub-classes and attributes to describe them. In this paper, we use the ontology language based on description logic (OWL DL) to represent the classes in the model. For example, using the description logic to describe the affected *Product*:

*Product*⊆*Platform* ∩

  ∀*hasPlatformType.PT* (*Application*∪*Hardware*∪*OperatingSystem*)

  ∃*hasSupplyChain. String ∩*

  ∀*hasVendor. String* ∩

  ∀*hasVersion. Version* ∩

  ∀*hasStatus. Status* (*Normal Vulnerable*∪*Serious Vulnerable* ∪ *Critical Vulnerable*)

#### 4.1.2. Ontology Description of the Vulnerability

In IoTSTO, the CVSS score describes the possible impact of the *Vulnerability* instance and also evaluates the severity of the *Vulnerability*. Taking vulnerability CVE-2017-7921 as an example—this vulnerability occurs when an application does not adequately or correctly authenticate users. Attackers can use token impersonation or session hijacking to escalate his or her privileges and gain access to sensitive information. CVE-2017-7921 is an instance of the *Vulnerability*, which is described as follows:

*Vulnerability* (CVE-2017-7921)∩

  ∀*hasSeverity. Severity* *(Critical* *) ∩*

  ∀*hasAttackVector. AV* (*Network*) *∩*

  ∀*hasAttackComplexity. AC* (*Low*) *∩*

  ∀*hasPrivilegesRequired. PR* (*None*) ∩

  ∀*hasUserInteraction. UI* (*None*) ∩

  ∀*hasScope. S* (*Changed*) ∩

  ∀*hasConfidentiality. C* (*High*) ∩

  ∀*hasIntegrity. I* (*High*) ∩

  ∀*hasAvailability. A* (*High*) ∩

  ∃*exploitedBy. AttackPattern* (*Token Impersonation* ∪ *Session Hijacking*)

#### 4.1.3. Ontology Description of the Weakness

In this paper, we introduce the *Modes_Of_Introduction* to describe how and when to import this weakness into the IoT environment. The subclass *Phase* identifies points that may be imported into the product life cycle. The subclass *Note* provides typical scenarios related to a specific import phase. CWE abstracts the weaknesses into 10 categories according to the relationship between them. *Weakness_Type* in this paper adopts this kind of classification method. *Improper Authentication* is an instance of the *Weakness*. When an attacker claims to have a given identity, the software does not prove or insufficiently proves that the claim is correct or authentic, which is described as follows:

*Improper Authentication* ⊆*WeaknessType (Improper Access Control)*⊆*Weakness* ∩

  ∀*hasCWE_ID. CWE_ID* (CWE-287) ∩

  ∀*hasApplicablePlatform. String* ∩

  ∀*hasWeaknessMitigation. String* ∩

  ∀*hasModesOfIntroduction. MOI* (*Phase ∩ Note*) *∩*

  ∀*hasLikelihoodOfExploit. LOE* (*High*)

#### 4.1.4. Ontology Description of the Attack Pattern

CAPEC divides an attack pattern into nine categories according to the mechanism used by the attacker when exploiting the vulnerability. In this paper, the *Attack Mechanism* adopts this classification method, which includes nine subclasses that correspond to the classification given by CAPEC. *Session Hijacking* is an instance of the *Attack Pattern*; this type of attack involves an attacker that exploits the weaknesses in an application’s use of sessions when performing authentication. The attacker is able to steal or manipulate an active session and use it to gain unauthorized access to the application, which is described as follows:

*AttackMechanism* (*Session Hijacking*) ⊆ *AttackPattern* ∩

  ∀*hasCAPEC_ID. CAPEC_ID* (CAPEC-593) ∩

  ∀*hasAttackLikelihood. AL* (*High*) ∩

  ∀*hasAttackPatternMitigation. String* ∩

  ∀*hasConsequence*. *C* (∃*Scope* ∩ ∃*Impact*) ∩

  ∀*hasPrerequisite. String* ∩

  ∀*hasResourcesRequired. String*

#### 4.1.5. Ontology Description of the Campaign

Compared with an attack, a campaign is a set of malicious activities or attacks that occur over a period of time against a specific set of targets. A *Campaign* class is a formal description of the tactics and techniques used by the attack group. In the ATT&CK framework, each tactic contains multiple techniques, and each technique is composed of multiple sub-techniques. The ontology modeling of this paper refers to its structure. *Man-In-The-Middle* technique refers to the attackers positioning themselves between two or more networked devices to support follow-on behaviors such as Network Sniffing or Transmitted Data Manipulation. *Man-In-The-Middle* is an instance of the *Technique*, which is described as follows:

*Technique* (*Man-in-the-Middle*) ⊆ *Campaign* ∩

  ∀*belongToTactic*. *TA* (*Credential Access* ∩ *Collection*) ∩

  ∀*hasSubTechnique. SubT* (*LLMNR/NBT-NS Poisoning and SMB Relay ∩ ARP Cache*

  *Poisoning*) ∩

  ∀*hasMitigation. String* ∩

  ∃*hasSoftware.* (*Tool ∩ Malware*) *∩*

  ∀*hasThreatGroup. Group*

### 4.2. Rule of Inference Design

In this paper, we choose Semantic Web Rule Language (SWRL) [44] to deal with the direct and indirect relationship of the ontology model to enhance the description ability of OWL. SWRL and OWL are based on the same description logic. SWRL has stronger expressive ability in solving the multi-level and complex interrelational reasoning and data value reasoning in ontology [45]. SWRL can directly use the relationships and vocabulary described in the ontology when writing rules of inference. Each SWRL rule is an OWL axiom in the ontology, and these new rules can also interact with the existing axioms in the ontology. The form of the SWRL is given as follows:

$$A_{1}{,\ldots ,\;A}_{m}\;\to {\;B}_{1}{,\ldots ,\;B}_{n}$$

**Figure 4:**
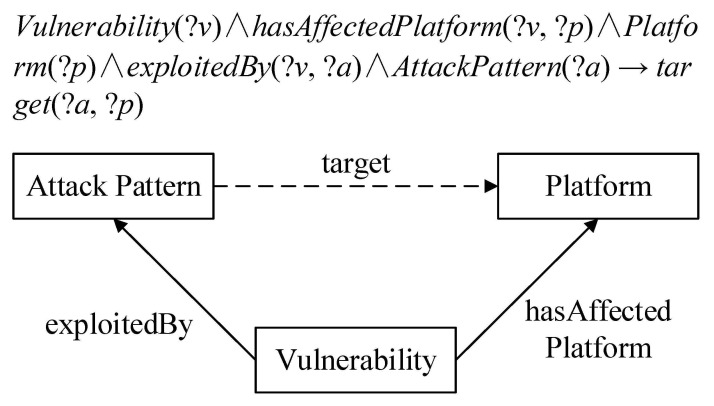
Examples of the SWRL inference rule and representation.

The commas on both sides of the arrows indicate conjunctions, which can be written as a conjunctive form and a disjunctive form when describing complex logical relationships.${\;A}_{1}{,\ldots ,\;A}_{m}\;\to {\;B}_{1}{,\ldots ,\;B}_{n}$ can be expressed as C(x), P(x, y), or (x, y). Here, *C* is an OWL description, *P* is an OWL property, and *x* and *y* can be datalog variables, OWL instances, or OWL data values. The rule of inference can discover new implicit knowledge from explicit knowledge. The following example shows the usage of the SWRL rule. Figure 4 reveals the new relationship according to the inference rule.

In the inference rule above, the object properties *hasAffectedPlatform*(?*v*, ?*p*) provides the relationship linkage between the class *Vulnerability*(?*v*) and the class *Platform*(?*p*). The object properties *exploitedBy*(?*v*, ?*a*) provides the relationship linkage between the class *Vulnerability*(?*v*) and the class *AttackPattern*(?*a*). This kind of relational linkage can infer implicit facts from the object properties *target*(?*a*, ?*p*) and existing knowledge.

The SWRL cannot make the OWL query. Therefore, we use Semantic Query-Enhanced Web Rule Language (SQWRL) to perform knowledge retrieval on the integrated ontology model. SQWRL is an extension of SWRL, and it can be used in conjunction with SWRL to transform existing rules into a pattern-matching mechanism. It allows for the query and retrieval of implicit knowledge inferred from OWL classes, OWL object properties, OWL data properties, and OWL individuals. The example in Section 4 shows the specific usage of inference rule in the multi-source knowledge inference model of IoT security.

## 5. Examples and Evaluation

In this section, we demonstrate several scenarios to further illustrate the feasibility and effectiveness of the proposed model. As for the coalescence and modeling of the multi-source information security knowledge base, the first subsection provides a linkage example to demonstrate the ability of the integrated graph data in order to provide context semantic information. The second subsection focuses on the design of inference rules based on the multi-source knowledge of IoT security. Various hardware and software in the IoT correspond to instances in the ontology. The security status of the IoT environment is reflected in the ontology. In this paper, instances in the IoT environment are mapped in the ontology, and the ability of the model to construct threat assessments of the IoT environment is demonstrated by its design of inference rules.

### 5.1. Linkage Example and Feasibility Analysis

This paper integrates multiple cyber security knowledge bases to provide a seamless set of paths that connect them. In order to demonstrate the feasibility and advantages of this method, this section uses a linkage query as an example, such as that of “video surveillance devices” to “Privilege Escalation” and “Defense Evasion” tactics. The entries in the linkage are as follows:

- *Tactic* (TA0004) *Privilege Escalation*: This adversary is trying to gain higher-level permissions. Privilege Escalation consists of techniques that adversaries use to gain higher-level permissions in a system or network. Adversaries can often enter and explore a network with unprivileged access but require elevated permissions to follow through on their objectives. Obtaining an account that is necessary for attackers to achieve their goals of gaining access to a specific system or performing a specific authorized operation can also be considered a privilege escalation. Common approaches are taking advantage of system weaknesses, misconfigurations, and vulnerabilities.
- *Tactic* (TA0005) *Defense Evasion*: This adversary is trying to avoid being detected. Defense Evasion consists of techniques that adversaries use to avoid detection throughout their compromise. Adversaries also leverage and abuse trusted processes to hide and masquerade their malware.
- *Technique* (T1134) *Access Token Manipulation*: Adversaries may modify access tokens to operate under a different user or system security context to perform actions and bypass access controls. The operation system, such as Windows, uses access tokens to determine the ownership of a running process. A user can manipulate access tokens to make a running process appear as though it is the child of a different process or belongs to someone other than the user that started the process.
- *Attack Pattern* (CAPEC-633) *Token Impersonation*: An adversary exploits a weakness in authentication to create an access token that impersonates a different entity, and then associates a process to that that impersonated token. Attackers can use this operation to use tokens to verify identity and take actions based on that identity.
- *Weakness* (CWE-287) *Improper Authentication*: When an actor claims to have a given identity, the platform does not prove or insufficiently proves that the claim is correct.
- *Vulnerability*: CVE-2017-7921. The improper authentication vulnerability occurs when an application does not adequately or correctly authenticate users. This may allow a malicious user to escalate his or her privileges on the system and gain access to sensitive information.
- *Affected platform* and CPE: “cpe:2.3:o: hikvision:ds-2cd2032-i_firmware:-:*:*:*:*:*:*:*’,” According to the CPE entry, the affected platforms are Hikvision video surveillance devices with firmware version DS-2CD2032-I.

The description of the linkage above is based on tactic from the perspective of attackers, assuming that the attacker’s goal is to obtain higher-level permissions without being detected. That is, by manipulating the access token to run as different users or in different systems in order to perform operations and bypass access control. Attackers can exploit the vulnerability of the Hikvision video surveillance devices, whose firmware version is DS-2CD2032-I, to simulate the access tokens of different entities through the weaknesses in authentication, and then escalate the privilege to obtain sensitive information and control this video surveillance device.

On the other hand, the description of the linkage is based on the affected platform from the perspective of defenders. If there are Hikvision video surveillance devices with the firmware version DS-2CD2032-I in cyberspace, network administrators need to be vigilant of attackers simulating access tokens through weaknesses in authentication, bypassing access control, and achieving privilege escalation. Network administrators can restrict permissions of users and user groups who cannot create tokens, or define token permissions for specific users in order to manage and restrict token creation. At the same time, network administrators can restrict users and accounts to the minimum privileges they need. They can reduce the path where attackers can bypass access control and narrow the possible attack surface to mitigate threats.

**Figure 5:**
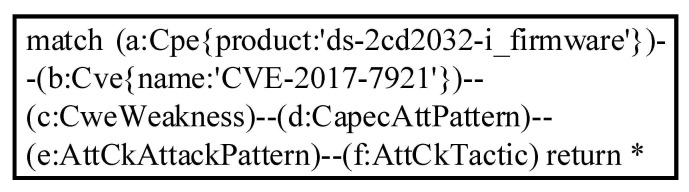
Cypher query for CVE-2017-7921.

As shown in the example above, researchers who use this method to integrate multi-source knowledge bases can query context information from different knowledge bases with any entry in the link set given. In fact, this method can traverse the knowledge graph to achieve more powerful query functions according to specific query requirements. The preprocessed graph data is stored in Neo4j. We used the query language Cypher and queried the Hikvision video surveillance device with the firmware version DS-2CD2032-I and the vulnerability entry CVE-2017-7921. The Cypher query statement is shown in Figure 5.

**Figure 6:**
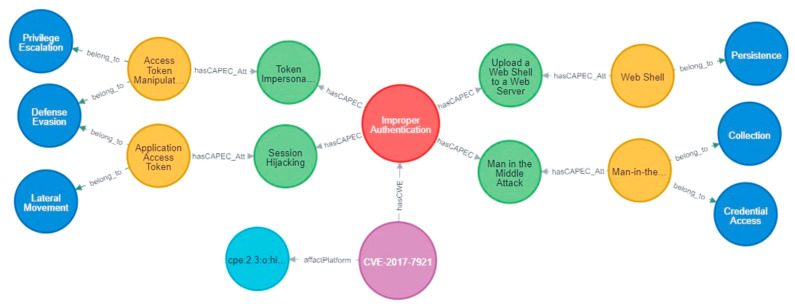
Cypher query result visualization for CVE-2017-7921.

Figure 6 shows the visualization of the query results. After the vulnerability CVE-2017-7921 is associated with the weakness CWE-287 Improper Authentication, four linkages associated with CWE-287 are traversed in the graph database, with the sample linkage query above also being among them. The context information contains several public security information resources that have been published and associated. The integration of multi-source knowledge existing in a specific IoT environment can improve the analysis ability and comprehensibility of CTI.

### 5.2. Inference Rules Based on Multi-Source Knowledge of IoT Security

**Figure 7:**
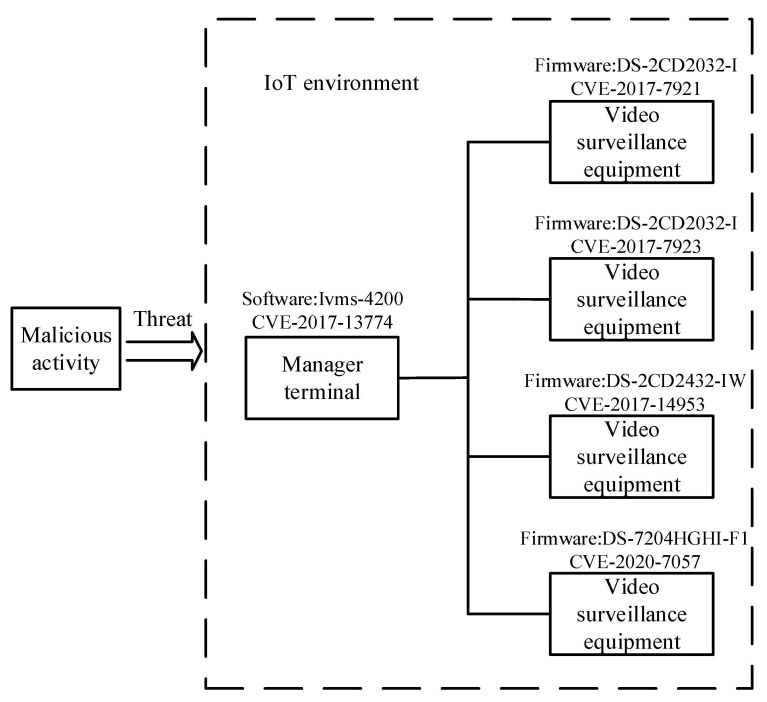
IoT context example.

This paper constructs a sample scenario for IoT testing, as shown in Figure 7. All components are instantiated in the IoTSTO. There is a video surveillance device collaboration group in the IoT, and devices are all connected to the Internet. There are multiple Hikvision video surveillance devices. The firmware versions are DS-2CD2032-I, DS-2CD2432-IW, and DS-7204HGHI-F1. The manager of the video surveillance device collaboration group uses the Ivms-4200 network video surveillance software. The existing vulnerabilities in the environment and their CVSS scores and severity are shown in Table 2.

**Figure 8:**
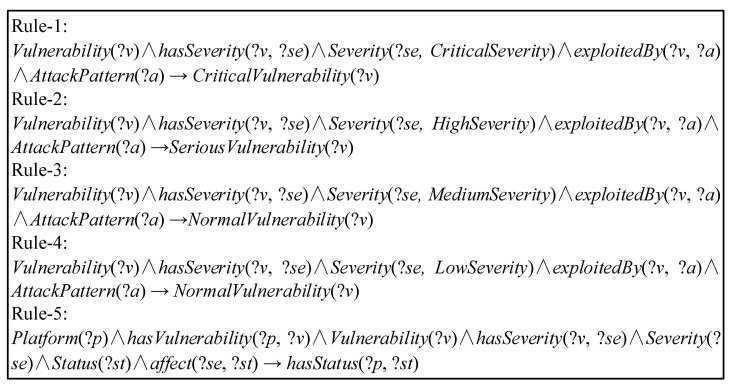
Reasoning the security status of the IoT environment.

These components are instantiated in the IoTSTO. For this paper, we designed the five inference rules to infer the security status in the IoT environment, as shown in Figure 8.

**Figure 9:**
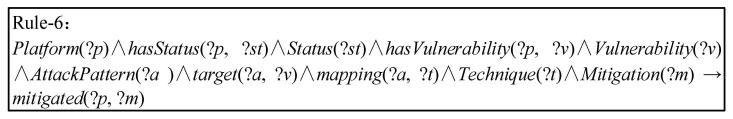
Reasoning mitigation.

Rule-1 to Rule-4 identify the *Severity* based on the known CVSS score of the *Vulnerability*. The semantics of Rule-5 is that there are *Platforms* affected by the *Vulnerability* in the IoT environment. When the *Severity* of the *Vulnerability* is “High” or “Critical”, the *Platform* affected by the *Vulnerability* in the system is *Critical Vulnerable* to malicious activities. The system infers the *Critical Vulnerable* areas of the *Platform* and provides support for security analysts to assess the *Severity* of threats to the IoT environment. Based on the inference rules above, we designed Rule-6 to realize automatically inferred *Mitigations* for *Critical Vulnerable Platforms*, as shown in Figure 9.

The semantics of this rule is that when the location of the *Critical Vulnerable* in the IoT environment is known, the *Attack Pattern* that may be subject to the *Vulnerability* in the *Platform* is mapped to the *Technique*. Then, network managers use the known threat events in the knowledge base to analyze the malicious activities and tactics associated with the specific *Technique*. Finally, the inference engine automatically infers the *Mitigations* that can be adopted in the system.

**Figure 10:**
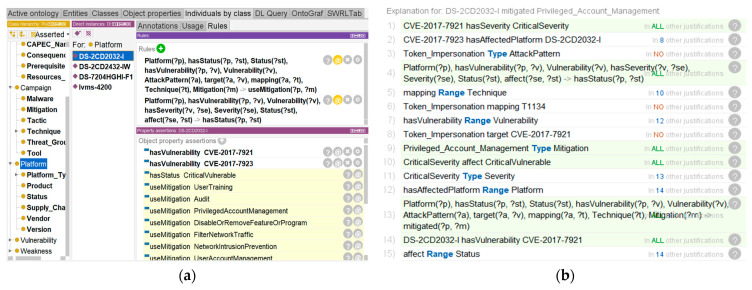
(**a**) Results from the application of inference rules; (**b**) The joint inference process.

After constructing the rules above, the Pellet reasoner is initiated for reasoning. According to the DS-2CD2032-I entity, new implicit facts are inferred. The *Status* of DS-2CD2032-I is classified as *Critical Vulnerable* and is associated with *Mitigation* through the object property *useMitigation*. The reasoning result is shown in Figure 10a.

**Figure 11:**
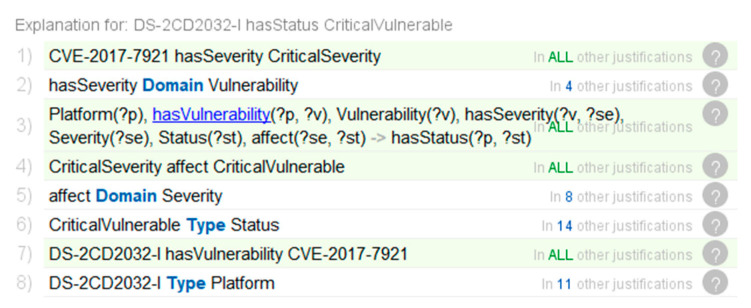
Inference process to infer the security status.

Through the interpretation function of Protégé, we showed the inference process of the security status of the IoT environment, and demonstrated the process of Rule-6 of automating inference mitigations inferred by the implicit facts. The details are shown in Figure 10b and Figure 11.

The detailed inference processes are described as follows:

1. A video surveillance device with firmware DS-2CD2032-I is deployed in the IoT. According to the explicit knowledge in the knowledge bases CVE and NVD, this video surveillance device has a CVE-2017-7921 vulnerability, and the *Severity* is *CriticalSeverity*;
2. System classifies the *Status* describing the vulnerability of the devices as *CriticalVulnerable*, which is based on the *Severity* of the *Vulnerability* associated with the video surveillance device
3. According to the explicit knowledge in the knowledge base CAPEC and the ATT&CK matrix, the *Attack Pattern* Token Impersonation is mapped to *Technique* T1134. System analyzes related threat events, which can infer appropriate *Mitigations* to mitigate threat activities that may be generated by adversaries.

**Figure 12:**
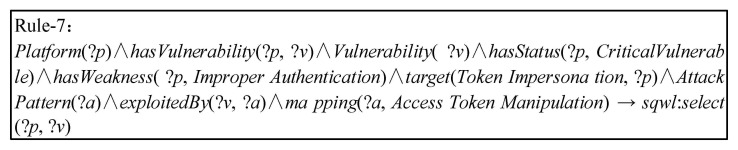
Separate critical vulnerable platforms with specific patterns.

The inference engine can separate the IoT nodes that are subjected to specific threats. The weaknesses and threats in the IoT environment are random and large in number. However, the targets of some threats are specific, and the characteristics of threat activities are obvious [46]. After perceiving the distribution of vulnerability in the IoT environment, the system combines the context information of threat intelligence to separate high-risk nodes and prioritize the processing of specific threats, which will greatly reduce spatio-temporal consumption and increase the speed of the response to threats [47,48]. For example, as shown in Figure 12, Rule-7 checks whether there are weaknesses of *Improper Authentication* in the IoT environment. The inference engine separates high-risk nodes that may be used by attackers in order to use the technique *Access Token Manipulation* to complete *Token Impersonation* of the IoT environment. In addition, network managers only need to add corresponding instances to the data layer in order to update the *Platforms* in the IoT environment, while IoTSTO and rule of inference can easily infer the security *status* in the IoT.

The example verifies the ability of our method to discover and automatically defend against threats in a heterogeneous IoT environment. If the system has vulnerabilities that are exploited by certain attack patterns, tactics, or techniques, the IoTSTO will perceive the critical-risk locations of system security through preset inference rules. Security managers can automatically infer the available mitigations and separate IoT nodes with specific threats from the constructed multi-source knowledge base. This method provides support for security managers to perceive the overall security situation and deploy the appropriate security solutions. The most important thing is to design the inference rules. Inference rules should be designed according to the actual IoT environment, and the characteristics of the complex IoT environment need to be accumulated and analyzed.

The current cyber security ontology models focus on different scopes of information. Some models focus on the integration of IoT assets, vulnerabilities, and weaknesses [19,43,49], and some models focus on modeling attack in the IoT environment [12,18,20,39,50,51]. Our proposed ontology model focuses on the information on IoT assets, vulnerabilities, weaknesses, attack patterns, techniques, and tactics, which gives a holistic view of the cyber security situation and is more comprehensive than the other models. Table 3 compares IoTSTO and other ontology models to show the scope of the knowledge domains involved in each cyber security ontology.

In this paper, IoTSTO focused on the affected platform, vulnerability, weakness, attack pattern, tactic, and technique used by attackers in the IoT, and provided a broader perception of the security status of the IoT. The scalability of IoTSTO is sufficient to accommodate the rapid transformation of the IoT architecture. Managers can define relevant inference rules based on the characteristics of the observed IoT environment in order to meet the adaptability of the model to the actual IoT environment and enhance the inference ability of the ontology.

## 6. Conclusions

In this paper, we integrated vulnerabilities, weaknesses, affected platforms and tactics, attack techniques, and attack patterns into a coherent set of links. This method enriched the context information of the network security knowledge base, which improve the analytical ability and comprehensibility of the CTI. We resolved the semantic heterogeneity problem by facilitating the formalization of knowledge in the IoT domain. In addition, we proposed an IoT security threat ontology model to describe the elements of IoT security threats, and we used a Pellet reasoner and an inference rule to perceive threats in a complex heterogeneous environment.

However, our work is not enough to monitor the overall security of the IoT. Subsequently, we will add cyber supply chain information to the ontology model. For example, we can add the supply chain information of intelligent manufacturing devices, such as third party vendors, suppliers, inbound supply chain, outbound supply chain, and other status information to the ontology model. In this way, it will not only detect the threat, but will also be able to detect the novel supply chain attacks by fusing the supply chain information of the intelligent manufacturing physical system with the cyber security information.

## Figures and Tables

**Table 1:** Level 2 and level 3 ontology class definitions.

Level	Class	Level	Class
2	Platform	3	Status, Platform_Type, Product, Supply_Chain, Vendor, Version
2	Vulnerability	3	CVSS, Impact, Severity
2	Weakness	3	CWE_ID, Modes_Of_Introduction, Weakness_Type, Applicable_Platform, Weakness_Mitigation, Likelihood_Of_Exploit
2	Attack Pattern	3	CAPEC_ID, Attack_Likelihood, Attack_Mechanism, Attack_Pattern_Mitigation, Consequence, Prerequisite, Resources_Required
2	Campaign	3	Malware, Mitigation, Tactic, Technique, Threat_Group, Tool, Sub-Technique

**Table 2:** Vulnerability, CVSS score and severity.

IoT Platform	Vulnerability	CVSS V3	Severity
DS-2CD2032-I	CVE-2017-7921	10.0	Critical
DS-2CD2032-I	CVE-2017-7923	8.8	High
Ivms-4200	CVE-2017-13774	7.8	High
DS-2CD2432-IW	CVE-2017-14953	6.5	Medium
DS-7204HGHI-F1	CVE-2020-7057	5.3	Medium

**Table 3:** Knowledge domain scope of several related cybersecurity ontology models.

	Platform	Vulnerability	Weakness	Attack Pattern	Technique	Tactic
UCO [12]		√		√	√	
MALOnt [18]		√		√	√	
IoTSec [19]	√	√	√			
PIoTCO [20]	√			√		
NSSA [39]	√	√		√		
VulKG [43]	√	√	√			
VCO [49]	√	√	√	√		
SVO [50]		√	√	√		
SKO [51]		√		√		
IoTSTO	√	√	√	√	√	√

## Data Availability

Not applicable.
